# Supplementary material for: Predicting the Landscape Epidemiology of Foot-and-Mouth Disease in Endemic Regions: An Interpretable Machine Learning Approach
Source: Viruses. 2025 Oct 17;17(10):1383. doi: 10.3390/v17101383 (PMC12567987; doi:10.3390/v17101383)
Supplement: Supplementary file 1 [file viruses-17-01383-s001.zip › viruses-3908777-supplementary.pdf]

Predicting the Landscape Epidemiology of Foot-and-Mouth Disease in Endemic Regions: An Interpretable Machine Learning Approach

Moh A. Alkhamis, Hamad Abouelhassan, Abdulaziz Alateeqi, Abrar Husain, John M. Humphreys, Jonathan Arzt, Andres M. Perez

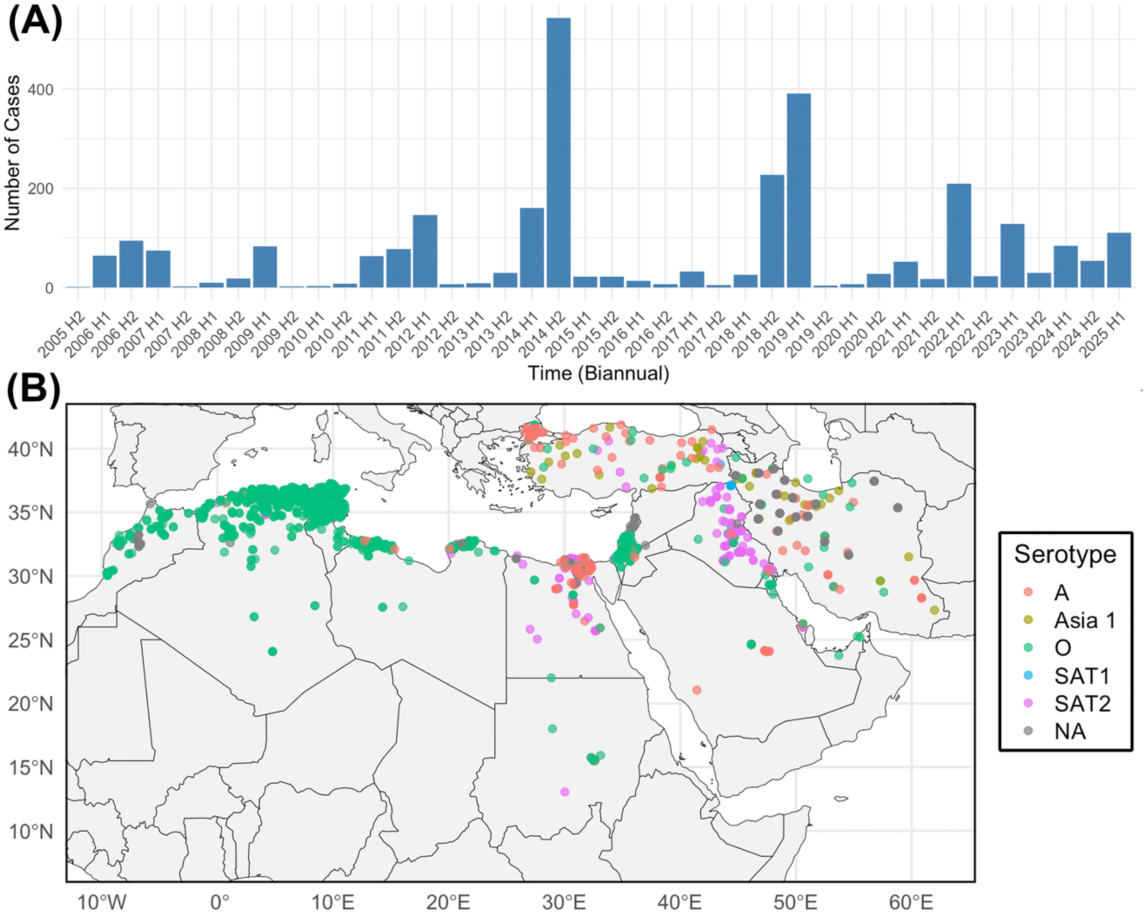

Figure S1. Spatial and temporal distribution of Foot-and-Mouth Disease (FMD) outbreaks in the Middle East and Northern Africa between 2005 and 2025 (N=2884). (A) temporal distribution of the outbreaks on a biannual basis. H1 and H2 represent 1st and second halves of the corresponding year, respectively. (B) Spatial distribution of the outbreaks, color-coded by serotypes. NA represents the undefined serotype of a reported FMD outbreak

Table S1. Ecological data source and attributes.

Feature	Source	Resolution	Year/Version
Annual mean temperature<br>Isothermality<br>Mean diurnal range<br>Temperature seasonality<br>Maximum temperature of the warmest month<br>Minimum temperature of the coldest month<br>Annual temperature range<br>Mean temperature of the warmest quarter<br>Mean temperature of the coldest quarter<br>Annual precipitation<br>Precipitation of the wettest month<br>Precipitation of the driest month<br>Precipitation seasonality<br>Precipitation of wettest quarter<br>Precipitation of driest quarter<br>Average wind speed<br>Wind speed<br>Elevation	WorldClim Global Climate Data database (https://www.worldclim.org/data/worldclim21.html)	5 min. arc	1970-2000/ 2.1
Buffalo density<br>Cattle density<br>Goat density<br>Sheep density	FAO GeoNetwork (http://www.fao.org/livestock-systems/global-distributions/en/)	10 min. arc	2020/4.0
Global land cover<br>Artificial surfaces<br>Cropland<br>Grassland<br>Tree covered areas<br>Shrubs covered areas<br>Herbaceous vegetation<br>Mangroves<br>Sparse vegetation<br>Bare soil<br>Snow and glaciers<br>Water bodies	https://www.fao.org/land-water/land/land-governance/land-resources-planning-toolbox/category/details/en/c/1036355/	30 sec. arc	2014/1.0
Global ruminant production systems	FAO GeoNetwork (https://www.fao.org/livestock-systems/production-systems/en/)	10 min. arc	2018/5.0
Human population density	FAO GeoNetwork (http://geonetwork.fao.org/geonetwork/loc/en/html/fao_core_datasets.html#)	2.5 min. arc	2015/1.0
Road density	GRIP global roads database (https://www.globio.info/download-grip-dataset)	5 min. arc	2018/4.0

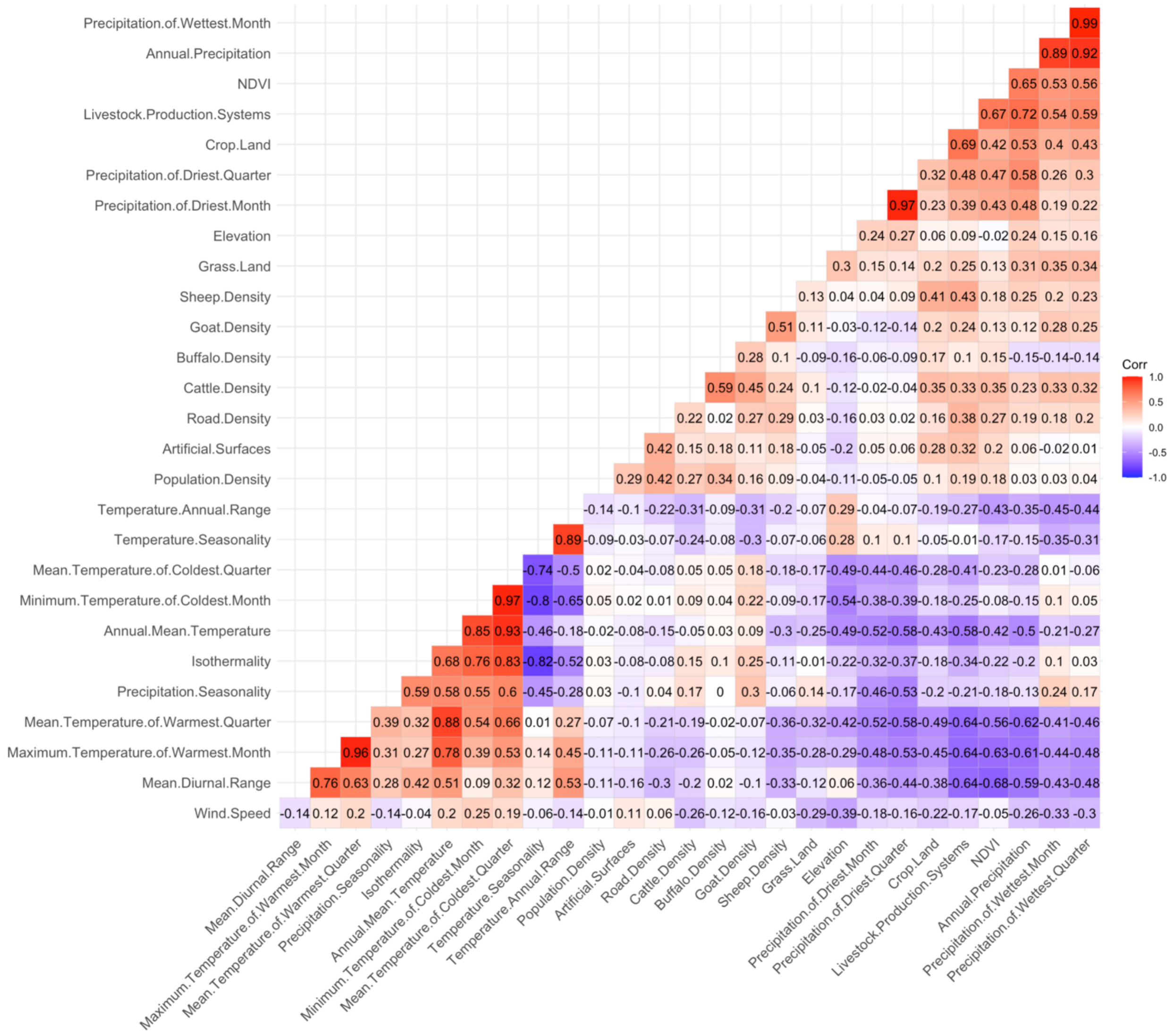

Figure S2: Correlation coefficient heatmap for features included in all models. Features where ρ > 0.9 were already excluded from the dataset. N.D.V.I = Normalized Difference Vegetation Index.
